# Multidimensional analysis of behavior predicts genotype with high accuracy in a mouse model of Angelman syndrome

**DOI:** 10.1038/s41398-022-02206-3

**Published:** 2022-10-03

**Authors:** Joseph K. Tanas, Devante D. Kerr, Li Wang, Anika Rai, Ilse Wallaard, Ype Elgersma, Michael S. Sidorov

**Affiliations:** 1 Center for Neuroscience Research, Children’s National Hospital, Washington, DC USA; 2 Howard University, Washington, DC USA; 3 Department of Clinical Genetics and the ENCORE Expertise Center for Neurodevelopmental Disorders, Erasmus MC, Rotterdam, Netherlands; 4 Departments of Pediatrics and Pharmacology & Physiology, The George Washington University School of Medicine and Health Sciences, Washington, DC USA

**Keywords:** Learning and memory, Autism spectrum disorders

## Abstract

Angelman syndrome (AS) is a neurodevelopmental disorder caused by loss of expression of the maternal copy of the *UBE3A* gene. Individuals with AS have a multifaceted behavioral phenotype consisting of deficits in motor function, epilepsy, cognitive impairment, sleep abnormalities, as well as other comorbidities. Effectively modeling this behavioral profile and measuring behavioral improvement will be crucial for the success of ongoing and future clinical trials. Foundational studies have defined an array of behavioral phenotypes in the AS mouse model. However, no single behavioral test is able to fully capture the complex nature of AS—in mice, or in children. We performed multidimensional analysis (principal component analysis + *k*-means clustering) to quantify the performance of AS model mice (*n* = 148) and wild-type littermates (*n* = 138) across eight behavioral domains. This approach correctly predicted the genotype of mice based on their behavioral profile with ~95% accuracy, and remained effective with reasonable sample sizes (*n* = ~12–15). Multidimensional analysis was effective using different combinations of behavioral inputs and was able to detect behavioral improvement as a function of treatment in AS model mice. Overall, multidimensional behavioral analysis provides a tool for evaluating the effectiveness of preclinical treatments for AS. Multidimensional analysis of behavior may also be applied to rodent models of related neurodevelopmental disorders, and may be particularly valuable for disorders where individual behavioral tests are less reliable than in AS.

## Introduction

Rodent models have enabled mechanistic insights into the genetic causes and circuit-level manifestations of single-gene neurodevelopmental disorders (NDDs) [1–7]. As mechanism-based treatments are developed for NDDs, the effectiveness of such treatments is often first tested preclinically by assessing improvements in mouse behavior. Behavioral phenotypes span multiple domains in individuals with NDDs (e.g., cognitive, motor, seizures, sleep), and a wide range of corresponding behavioral assessments have been developed and deployed in mouse models [8–11]. Accurately measuring phenotypic severity across multiple behavioral domains is critical to properly assess the effectiveness of treatments in rodent models of NDDs.

We hypothesized that multidimensional analysis of mouse behavior would enable quantification of overall behavioral severity, aggregated across multiple domains. Here we define multidimensional analysis as the multi-step process of: (a) reducing the dimensionality of large behavioral datasets using principal component analysis (PCA) [12, 13], (b) clustering data in principal component space using k-means clustering, and (c) assessing whether behaviorally defined clusters align with animal genotype. Dimensionality reduction and clustering have been validated in various mouse behavioral contexts [14–22]. Here, we tested the hypothesis that multidimensional analysis of mouse behavioral data could accurately distinguish the genotype of *Ube3a* mutants (a model of Angelman syndrome (AS)) from wild-type littermates. AS is an ideal disorder for testing the effectiveness of multidimensional analysis because of recent progress in developing mechanism-based treatments [23] and because behavioral testing can be performed reliably in Angelman model mice [24].

AS is a NDD caused by lack of expression of the maternal allele of *UBE3A*, an E3 ubiquitin ligase located on chromosome 15 [25–27]. Individuals with AS have a multifaceted behavioral phenotype that typically includes cognitive impairment, motor impairment, lack of speech, seizures, and disrupted sleep [28–30]. While mutations in maternal *UBE3A* are sufficient to cause AS, the majority of cases (~70%) are caused by deletions of a region of maternal chromosome 15q11–13 spanning *UBE3A* and neighboring genes [29]. The paternal *UBE3A* allele is epigenetically silenced in neurons by expression of a *UBE3A* antisense transcript (*UBE3A-ATS*) [31, 32], and neuronal paternal *Ube3a* imprinting is conserved [33] in the mouse model of AS (*Ube3a*^*m-/p+*^) [34]. Multiple approaches have successfully unsilenced the dormant paternal *Ube3a* allele in mice [35–42], and one such approach (antisense oligonucleotides targeted to *UBE3A-ATS*) is currently in early-stage clinical trials (NCT04428281, NCT04259281, NCT05127226). Other AS treatments such as gene replacement therapy and targeting downstream processes are also in preclinical development [43]. Developing a pipeline to test new AS treatments in mice will be critical to evaluate their success, regardless of treatment mechanism. Recent work established a “gold standard” behavioral battery consisting of five tests (rotarod, open field, marble burying, nest building, forced swim) that are reliably impaired in *Ube3a*^*m-/p+*^ mice [24] and are sensitive to treatment [40, 44–46]. Using this battery, we hypothesized that multidimensional analysis: (a) would enable quantification of behavior across multiple domains as a single “severity score,” (b) that this severity score would be a reliable indicator of *Ube3a* genotype, and (c) that this severity score is sensitive to treatment.

## Methods and materials

### Animals

We performed multidimensional analysis using three mouse behavioral datasets. All datasets assessed behavior in male and female AS model mice (*Ube3a*^*m-/p+*^) [34] and wild-type littermate controls (WT; *Ube3a*^*m+/p+*^) with experimenters blind to genotype. For all datasets, experimental WT and *Ube3a*^*m-/p+*^ littermates were generated by crossing female *Ube3a*^*m+/p-*^ mice and male WT mice. Dataset 1 used experimental mice on an F1 hybrid 129S2-C57BL6/J background, and Datasets 2 and 3 used experimental mice on a congenic C57BL6/J background. All experimental protocols were conducted in accordance with the European Commission Council Directive 2010/63/EU (CCD approval AVD101002016791; Dataset 1), or were approved by the Institutional Animal Care and Use Committee (IACUC) of Children’s National Medical Center (Dataset 2). Dataset 3 contained only previously published data and no additional mouse experiments.

**Fig. 1:**
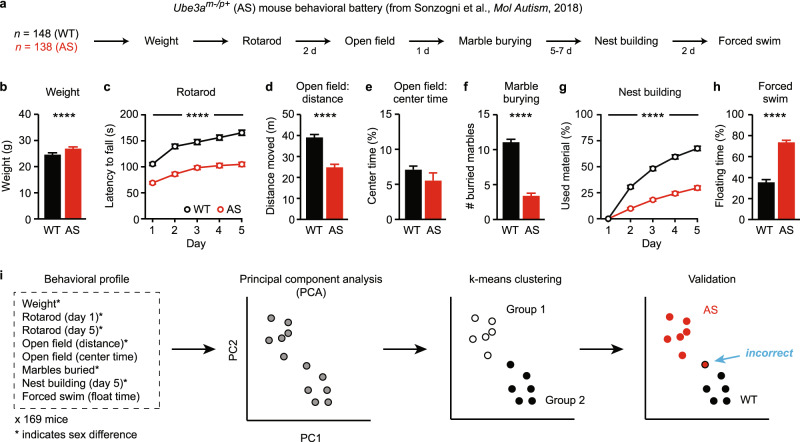
*Ube3a*^*m-/p+*^ mice have robust behavioral impairments across multiple domains. **a** Experimental timeline, as described and performed by Sonzogni et al. (eight cohorts), plus two additional unpublished cohorts. **b** Weight (WT: *n* = 118, AS: *n* = 110). **c** Rotarod performance (WT: *n* = 148, AS: *n* = 138). **d** Distance traveled and **e** time spent in the center of an open field (WT: *n* = 118, AS: *n* = 110). **f** Marble burying performance (WT: *n* = 148, AS: *n* = 138). **g** Nest building performance (WT: *n* = 109, AS: *n* = 100). **h** Forced swim performance (WT: *n* = 148, AS: *n* = 138). See Fig. S1 for full breakdown of tests performed in each of ten cohorts. Data represent mean ± SEM; ****p < 0.0001; black: WT, red: *Ube3a*^*m-/p+*^ (AS). **i** Methods for multidimensional behavioral analysis and genotype validation. First, eight behavioral measures were included in multidimensional analysis. Second, principal component analysis (PCA) reduces the dimensionality of the behavioral dataset. Each point represents one animal; these points are schematized and are not real data. Third, mice are clustered into two groups using k-means clustering by their proximity in PC space. Finally, validation reflects a comparison of clusters with the known genotype of animals.

**Fig. 2:**
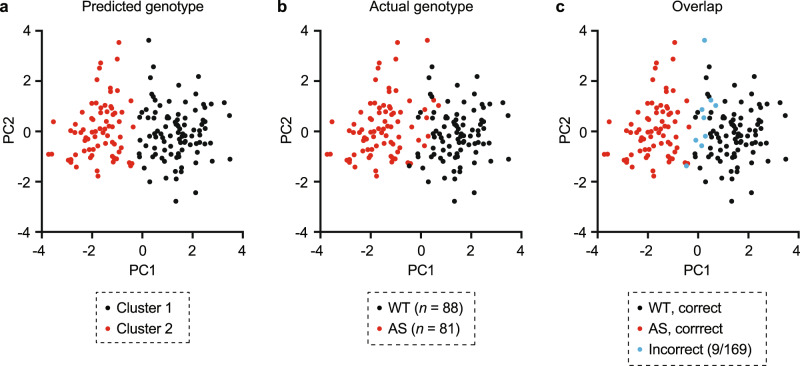
Multidimensional behavioral analysis predicts *Ube3a* genotype with high accuracy. **a** PCA plus k-means clustering categorized two clusters, with each dot representing one mouse’s behavioral profile in 2PC space (black: cluster 1, red: cluster 2). **b** Actual genotype of animals (black: WT, red: AS). **c** An overlay of **a**, **b** with animals clustered incorrectly labeled in blue. Clustering accuracy was 94.7%.

**Fig. 3:**
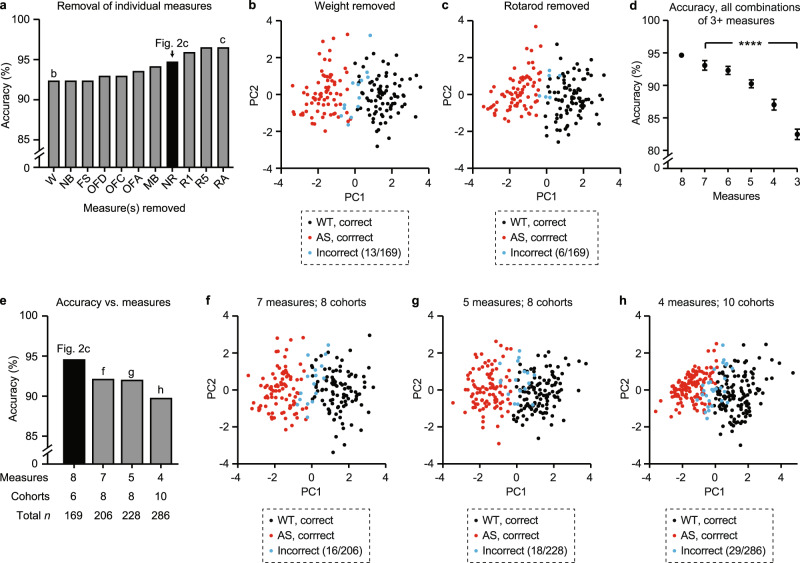
Multidimensional analysis remains effective with different combinations of behavioral data. **a** Removal of individual measures and individual behavioral tests resulted in clustering accuracies ranging from 92–97%. Each bar represents a condition where one measure or test was excluded; black bar represents the full dataset (*n* = 169) with nothing excluded. **b** The lowest accuracy achieved was 92.9% with weight excluded from analysis. **c** The highest accuracy achieved with removal of a single test was 96.5% with rotarod excluded. **d** Average accuracy across all combinations of measures used ranged from 82.5% (3 measures) to 93.1% (7 measures). Data represent mean ± SEM; ****p < 0.0001, one-way ANOVA. 8 measures: *n* = 1, 7 measures: *n* = 8, 6 measures: *n* = 28, 5 measures: *n* = 56, 4 measures: *n* = 70, 3 measures: *n* = 56. **e** Multidimensional analysis in larger sample sizes was achieved by including fewer behavioral tests. **f**–**h** Accuracy of different conditions shown in **e** ranges from 89.9% to 92.2%.

**Fig. 4:**
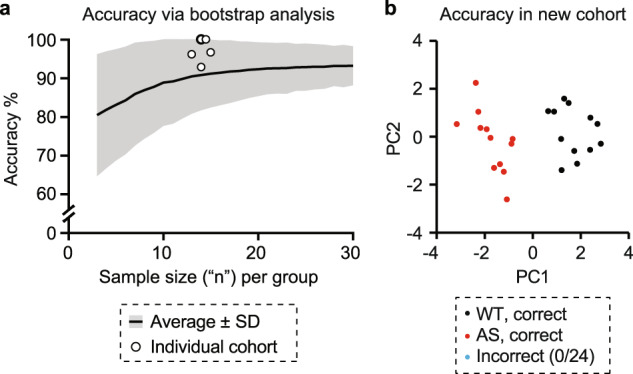
Multidimensional analysis retains high accuracy with reasonable sample sizes. **a** Bootstrap analysis was performed using Dataset 1, and 10,000 trials per *n*. Open circles indicate the clustering accuracy within six individual cohorts. The bold circle represents the overlap of two cohorts with the same (*x*,*y*) coordinates (14, 100%). **b** Clustering accuracy using Dataset 2 (*n* = 12 per genotype) was 100%.

**Fig. 5:**
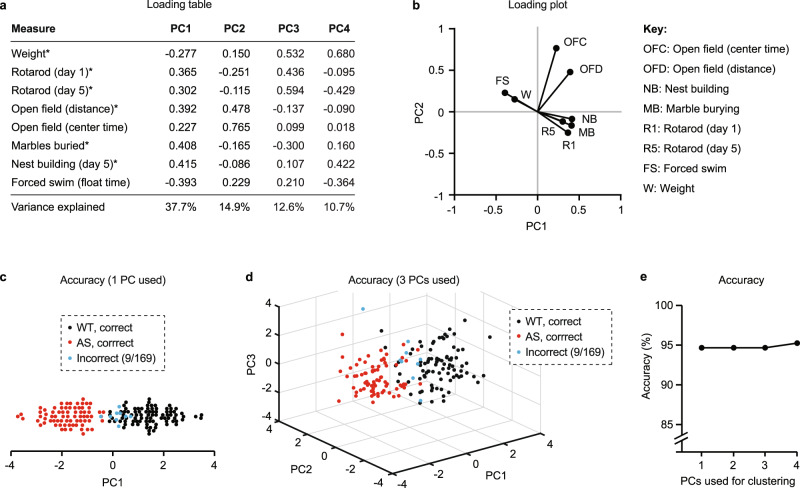
Principal component 1 (PC1) is sufficient for predicting *Ube3a* genotype with high accuracy. **a** Loadings for PC1–PC4 for each behavioral measure. **b** Loadings for PC1 and PC2 for each measure, plotted in 2PC space. All behavioral measures except open field correlate more strongly with PC1, and both open field measures correlate more strongly with PC2. **c** Clustering accuracy using 1 PC is 94.7%. **d** Clustering accuracy using 3 PCs is 94.7%. **e** Increasing the number of PCs used for clustering does not have a major impact on clustering accuracy.

Dataset 1 was used for the majority of analyses (Figs. 1–3, 4a, 5, S1–S8, and S10) to assess the effectiveness of multidimensional analysis at predicting *Ube3a* genotype based on behavior. Dataset 1 included 286 total mice (WT: *n* = 148, *Ube3a*^*m-/p+*^: *n* = 138) run across 10 cohorts at Erasmus Medical Center. Behavioral data from 8 of these 10 cohorts (*n* = 231/286 mice) were previously published [24] and 2 additional cohorts (*n* = 55 mice) were also included. A detailed table showing the genotypes, sex, and behavioral tests performed in each of the ten cohorts is shown in Fig. S1. Behavioral testing was performed in P60–P90 mice.

Dataset 2 was used to test the hypothesis that multidimensional analysis of behavior can effectively predict *Ube3a* genotype using small sample sizes (Fig. 4b and S9). Dataset 2 included 24 mice (WT: *n* = 12, *Ube3a*^*m-/p+*^: *n* = 12) tested at Children’s National Research Institute. Behavioral testing was performed in P60-P90 mice.

**Fig. 6:**
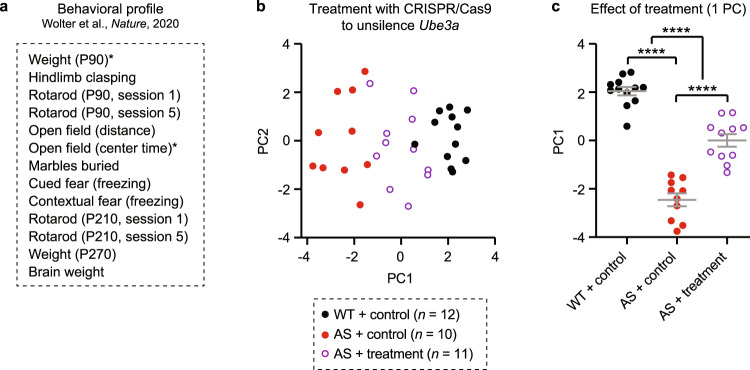
Multidimensional analysis has the sensitivity to detect behavioral improvement in *Ube3a*^*m-/p+*^ mice following treatment. **a** Multidimensional analysis used 12 behavioral measures from Wolter et al. (Dataset 3). *Indicates sex difference and standardization by sex. **b** Animals with paternal *Ube3a* unsilencing (purple open circles) have a qualitatively intermediate behavioral profile between *Ube3a*^*m-/p+*^ mutants (AS; red) and wild-type controls (WT; black). **c** In 1PC space, treated AS mice show overall behavioral improvement relative to control AS mice, but not full rescue. Full timeline of behavioral tests and analysis using different combinations of behavioral tests is shown in Fig. S11. Data represent mean ± SEM**;** ****p < 0.0001.

Dataset 3 was previously published (Wolter et al.; Supplementary Fig. 4) [41] and was used to assess whether multidimensional analysis could detect behavioral improvement in *Ube3a*^*m-/p+*^ mice treated with CRISPR-Cas9-based targeted treatment to unsilence the dormant paternal *Ube3a* allele (Fig. 6 and S11). Mice were treated with either viral expression of a SaCas9 gRNA targeting the *Ube3a-ATS* locus (Sajw33), or a negative control gRNA that did not unsilence paternal *Ube3a*. Bilateral i.c.v. AAV delivery of Sajw33 or control occurred at both E15.5 and P1 within the same animals, and behavioral testing began at 4 weeks and continued through 40 weeks [41]. Behavioral data included WT + control (*n* = 34), *Ube3a*^*m-/p+*^ + control (*n* = 25), and *Ube3a*^*m-/p+*^ + treatment (Sajw33; *n* = 32) groups.

### Behavioral testing

Mice in Datasets 1 and 2 were weighed and then underwent a series of behavioral tests in the same order: rotarod, open field, marble burying, nest building, and forced swim (Fig. 1a). A subset of mice in Dataset 1 lacked data from three of these tests (weight, open field, nest building; Fig. S1a). Methods for Dataset 1 were previously published [24] and methods for Dataset 2 (below) were based upon Sonzogni et al. [24], with minor modifications.

#### Rotarod

Mice were placed on a rotating bar that accelerated from 4 to 40 rpm across 5 min at an acceleration rate of 7.2 rpm^2^ (Ugo Basile model #47600). Trials were complete once the mouse fell off, if three consecutive wrapping rotations were made, or if 5 min elapsed. Each day, the results of two trials with an inter-trial interval of one hour were averaged together. Experiments were run over the span of five consecutive days, with a two day interval before open field testing.

#### Open field test

Mice were placed into a 42 cm square open field arena (AccuScan Instruments, Inc., Columbus, OH) and were allowed to freely move for a single 30 min trial. The center square was defined as 21 cm × 21 cm. The data was collected using the open field activity monitoring system (Omnitech Electronics, Inc. SuperFlex Open Field System) which uses photocell emitters and receptors forming an *x*–*y* grid of infrared beams. Total distance moved and time spent in the center square were recorded using infrared beam break information.

#### Marble burying

Mice were placed individually in a 16 × 8 in cage with ~4 cm of bedding (Bed-o’Cobs 1/4” bedding) and 20 black glass marbles arranged in a 5 × 4 array for a single 30 min trial. A marble was considered buried if it was >50% covered with bedding at the end of the trial.

#### Nest building

Immediately after marble burying, mice were habituated to single housing as well as new nesting material (Bio-Rad 7.5 × 10 cm extra thick block filter paper; 11 ± 1 g) for 5–7 days prior to testing. During testing, new nesting material was introduced on day 1 and unused material was weighed daily across five days.

#### Forced swim test

Forced swim testing was performed on the same day immediately following the final day of nest building. Mice were placed in a 9 in cylindrical × 9.25 in tall tank filled with 23 ± 1 °C water to a height of ~60% of the container’s height. Trials lasted 6 min: the first 2 min were an acclimation period and the last 4 min were to record immobility (lack of movement or only necessary movements to keep head above water) as a percentage of total recording time. Immobility was recorded manually using a stopwatch.

Mice in Dataset 3 underwent a different series of behaviors in consistent order: hindlimb clasping, rotarod, open field, marble burying, fear conditioning, rotarod (again) (Fig. S11a). Methods for Dataset 3 were previously published [41].

### Multidimensional analysis of mouse behavior

Multidimensional analysis of behavior consisted of a series of steps: data selection, standardization, principal component analysis (PCA), *k*-means clustering, and validation (Fig. 1i) [22]. Data selection: Eight total measures (weight, rotarod day 1, rotarod day 5, open field distance traveled, open field center time, marbles buried, nest building, forced swim floating time) from a series of six tests (weight, rotarod, open field, marble burying, nest building, forced swim) were included in multidimensional analysis. Tests with multiple measures include rotarod (day 1 and day 5 performance) and open field (total distance and time spent in the center of the arena). Measures considered redundant (e.g., intermediate, non-independent timepoints for rotarod and nest building) were excluded a priori from multidimensional analysis. For the majority of analyses (Figs. 2, 3a–d, 4a, 5, S3–S6, S8c, and S10), we included the subset of animals that underwent all six tests (WT: *n* = 88, *Ube3a*^*m-/p+*^: *n* = 82). One outlier was excluded from analysis based on its position in 2PC space (PC1: −2.49, PC2: 8.62) using Grubbs’ test for outliers (alpha = 0.05), leaving a total *n* = 81 *Ube3a*^*m-/p+*^ mice. Standardization: All measures were standardized using a *z*-score (*z* = (data point − group mean)/standard deviation) to account for different units across tests. Prior to standardization, we tested whether each behavioral measure showed significant sex differences using a two-way ANOVA with sex and genotype as factors (Fig. S2). All behaviors that showed either a significant main effect of sex or sex × genotype interaction were standardized separately in male and female animals (Fig. 1i), except where noted (Fig. S6; a separate analysis to assess the effectiveness of multidimensional analysis if sex is not accounted for). Measures with no sex differences were standardized using the entire group. Principal component analysis: We performed PCA using the pca() function in MATLAB and calculated the amount of variance explained by each PC using a Scree plot (Figs. S3, S9h, and S11b) and the loading distribution of principal components using the coefficient outputs from PCA (Figs. 5a, b and S11c). *k*-means clustering: *k*-means clustering was performed in principal component space using the kmeans() function in MATLAB with *k* = 2 clusters. Except where noted (Figs. 5, 6c, S4, and S11d, g), clustering was performed in 2PC space. In Figs. 5c, 6c, and S11d, g, one PC was used for clustering. Figure 5d, e evaluated using three and four PCs for clustering. In Fig. S4, clustering was performed on raw data (not in PC space). Validation: We compared the actual genotypes of animals to their assigned cluster and calculated the percentage correct (accuracy of clustering, Figs. 2c, 3b, c, f–h, 4b, 5c–e, S5a, S6, S10, and S11d).

In order to assess the generalizability of multidimensional analysis, we varied the input parameters of analysis in three ways (Fig. 3). First, we performed multidimensional analysis on Dataset 1 (*n* = 169; standardized by sex) in 2 PC space under 11 conditions (Fig. 3a–c). Each condition represented the removal of a single behavioral measure or a complete behavioral test. Next, we performed multidimensional analysis using every possible combination of 3–8 total measures (Fig. 3d). Finally, we performed multidimensional analysis on different combinations of data where more animals could be included with partial behavioral profiles (Fig. 3e–h and S1). This approach resulted in four conditions with the number of behavioral measures ranging from 4 to 8 and the number of animals ranging from 169 to 286 per condition.

To assess the minimal sample size needed for multidimensional analysis to effectively classify *Ube3a* genotype, we performed a bootstrap analysis of Dataset 1 (*n* = 169) using 2 PCs for clustering (Fig. 4a). For *n* = 3 to *n* = 30 with a step of *n* = 1, we randomly selected *n* animals per genotype from the overall dataset (*n* = 169), with replacement, and performed multidimensional analysis. We repeated 10,000 trials for each *n* and report the average clustering accuracy.

To determine whether multidimensional analysis results in false positive effects, we performed multidimensional analysis in a homogenous sample (all 36 wild-type females). We randomly assigned mice to two groups (to model two genotypes with no true behavioral difference; Fig. S7).

### Behavioral rescue with *Ube3a* reinstatement by Cas9 gene therapy

Behavioral data was available for a total of *n* = 91 mice in Dataset 3. However, every behavioral test was not performed in every animal. Thus, we performed multidimensional analysis in two subsets (“conditions”) of the total sample (Fig. S11e). Condition 1 (Fig. 6) included *n* = 33 total mice where behavioral data was available for each of thirteen total measures. Condition 2 (Fig. S11f, g) included *n* = 86 total mice where behavioral data was available for six of thirteen total measures. Data were standardized by sex for measures where statistically significant sex differences were observed between WT + control and AS + control groups in the total sample (P90 weight, open field center time).

### Statistics

Statistical analysis was performed using GraphPad Prism 9 and MATLAB R2019a, R2021a, and R2022a (Mathworks). Group comparisons on individual behavioral tests (Figs. 1 and S9) were made using Student’s t-tests (weight, open field distance, open field center time, marble burying, forced swim) and two-way RM ANOVA (rotarod, nest building). Assessment of sex differences (Figs. 2, S5c, and data not shown to accompany Fig. 6/S11) were made using two-way ANOVA. Effect of treatment was assessed using one-way ANOVA and post hoc Tukey’s multiple comparison test in 1 PC space (Figs. 6c and S11g). Figure 3d used one-way ANOVA and post hoc Tukey’s multiple comparison test. Figure S7d used Student’s *t* test. Fisher’s exact test was used to determine if clustering accuracy was statistically different between test conditions (Figs. 3a–c, S4, and S6). For all figures, *p < 0.05, **p < 0.01, ***p < 0.001, and ****p < 0.0001.

## Results

### *Ube3a*^*m-/p+*^ mice have robust behavioral impairments

Ten independent cohorts totaling 286 mice (Fig. S1; 26–30 mice per cohort) performed a series of behavioral tests in order: weight, rotarod, open field, marble burying, nest building, and forced swim (Fig. 1a). Behavioral data from eight of these cohorts (*n* = 231) were previously reported by Sonzogni and colleagues [24]. In the complete dataset, *Ube3a*^*m-/p+*^ mice showed increased weight (Fig. 1b; *t*_(226)_ = 4.428, *p* < 0.0001), impaired rotarod performance (Fig. 1c; main effect of genotype: *F*_(1,284)_ = 103.8, *p* < 0.0001), and impaired rotarod learning (Fig. 1c; genotype × time interaction: *F*_(4,1136)_ = 6.792, *p* < 0.0001) relative to WT controls. *Ube3a*^*m-/p+*^ mice were hypoactive in an open field (Fig. 1d; *t*_(226)_ = 8.874, *p* < 0.0001) despite normal time spent in the center of the arena (Fig. 1e; *t*_(226)_ = 1.348, *p* = 0.1789). *Ube3a*^*m-/p+*^ mice also showed impairments on marble burying (Fig. 1f; *t*_(284)_ = 16.79, *p* < 0.0001), nest building (Fig. 1g; main effect of genotype: *F*_(1,207)_ = 176.1, *p* < 0.0001), and forced swim tests (Fig. 1h; *t*_(284)_ = 15.73, *p* < 0.0001) relative to WT controls. Prior work found significant sex differences in a subset of these behaviors [24], and we confirmed with the complete dataset that six of eight behavioral measures showed statistically meaningful sex differences (Fig. S2).

### Multidimensional analysis of behavior predicts *Ube3a* genotype with high accuracy

From the group of six behavioral tests, we selected eight non-redundant measurements to include in multidimensional analysis (Fig. 1i). We performed multidimensional analysis using data from mice where all behavioral tests were performed within individual animals (*n* = 169/286; 6/10 cohorts**;** Fig. S1). Multidimensional analysis consisted of four steps: standardization, principal component analysis (PCA), *k*-means clustering, and validation (Fig. 1i). First, all measures were standardized using a *z*-score and measures with sex differences were standardized separately by sex. PCA revealed that two principal components (PCs) are likely sufficient to capture a majority of variance in the dataset (Fig. S3); thus, we performed *k*-means clustering in 2PC space (Fig. 2a). Validation comparing clusters (Fig. 2a) to the actual genotype of animals (Fig. 2b) demonstrated that multidimensional analysis was 94.7% accurate in predicting *Ube3a* genotype from behavior (Fig. 2c). Multidimensional analysis performed better than k-means clustering of performance on individual assessments, which ranged from ~55–85% accuracy (Fig. S4). Male and female mice were equally distributed in PC space (Fig. S5), confirming that sex differences in behavior were accounted for by standardization, and likely do not represent an additional source of meaningful variance. Not accounting for the sex dependence of behaviors slightly reduced the accuracy of multidimensional analysis (from 94.7% to 91.1%), though this difference was not statistically meaningful (Fig. S6). To confirm the validity of multidimensional analysis, we demonstrated that this approach does not detect “false positive” differences in behavior between two randomly assigned groups within a homogenous group of animals (all wild-type females) (Fig. S7).

### Multidimensional analysis remains effective with different combinations of behavioral input

For multidimensional analysis to be a valuable tool to quantify behavior in mouse models of AS and related disorders, it should generalize across multiple combinations of behavioral input. To address this question, we first performed multidimensional analysis on the same dataset (*n* = 169) under conditions where each individual measurement (e.g., rotarod day 5) or overall test (e.g., rotarod) were excluded from analysis. Removal of individual measures or tests resulted in a range of 92.3–96.4% accuracy, which was not statistically different from the baseline of 94.7% (Fisher’s exact tests; lowest *p* = 0.5092; Figs. 3a–c and S8a). Next, we assessed the clustering accuracy in each of 219 possible combinations of including between three and eight behavioral measures in analysis. Clustering accuracy decreased on average as the number of measures decreased (Fig. 3d; one-way ANOVA, *F*_(4,261)_ = 21.31, *p* < 0.0001). We also expanded the dataset to include all animals (*n* = 286), and defined four conditions where different combinations of behavior were available for different subsets of animals (Fig. S1). As the number of measures decreased, the accuracy of multidimensional analysis decreased despite the sample size increasing (Figs. 3e–h and S8b). Together, these data suggest that multidimensional analysis generalizes well across different combinations of behavioral data in the *Ube3a*^*m-/p+*^ mouse model, and gains effectiveness as more behavioral tests are included in analysis.

### Multidimensional analysis accurately predicts *Ube3a*^*m-/p+*^ genotype with reasonable sample sizes for mouse behavior

In practice, a behavioral study requiring >80 mice per genotype to detect group differences would likely be time and cost prohibitive. Here, we asked whether multidimensional analysis would retain high accuracy in groups with smaller sample sizes. To address this question, we performed a bootstrap analysis to predict the clustering accuracy that could be achieved across sample sizes ranging from *n* = 3–30 per group (Figs. 4a and S8c). We then performed multidimensional analysis on a behavioral cohort tested prospectively in a different laboratory with 12 animals per group (Dataset 2, Fig. S9). The bootstrap analysis predicted an accuracy of 90% using *n* = 12, and multidimensional analysis achieved 100% accuracy in this new cohort (Figs. 4b and S9). We hypothesized that accuracy was higher in a new cohort because the bootstrap analysis pulls animals randomly across multiple cohorts, introducing inter-cohort variability. We tested this hypothesis by performing multidimensional analysis separately in each of six individual cohorts from Dataset 1 (Fig. S9). Accuracy in each of these cohorts also outperformed the expectations of the bootstrap analysis, ranging from 92–100% with sample sizes of ~13–15 animals per genotype (Fig. 4a; open circles). The cohort sizes tested here are comparable to the sample sizes determined by Sonzogni et al. to be required to detect group level differences on individual behavioral tests (*n* = 7–21, depending on test) [24]. Overall, this work confirms that multidimensional analysis of behavior is highly accurate in *Ube3a* mice using typical sample sizes for mouse behavior.

### Multidimensional analysis remains effective using a single PC for clustering

A potentially valuable use of multidimensional behavioral analysis is the possibility of summarizing an animal’s overall phenotypic severity as a single number (PC1). Such an approach will only be valuable if (a) PC1 represents a substantial amount of the total variability in the dataset and (b) PC1 alone is sufficient to accurately predict *Ube3a* genotype. PC1 accounts for 37.7% of variance in the dataset (Figs. S3 and 5a) and correlates strongly with each behavioral measure tested (Fig. 5a, b). Clustering accuracy remained high (94.7%) using a single principal component for k-means clustering (Fig. 5c). Increasing the number of PCs to 3–4 (accounting for up to 75% of total variation) provided little additional benefit in predicting *Ube3a* genotype (Fig. 5d, e). Overall, these results suggest that PC1 alone is sufficient to accurately predict *Ube3a* genotype.

### Multidimensional analysis can detect behavioral improvement following paternal *Ube3a* unsilencing

Behavioral testing in mouse models of neurodevelopmental disorders is often used to test the preclinical effectiveness of treatments [10]. We performed multidimensional analysis of behavior in a group of mice where CRISPR/Cas9-based targeting of *Ube3a-ATS* enabled unsilencing of the paternal *Ube3a* allele and re-expression of UBE3A protein [41]. In this cohort (Dataset 3), the series of behavioral tests performed was similar but not identical to the series of tests performed by Sonzogni and colleagues [24] (Figs. 6a and S11a). In this dataset, PC1 represented 31.0% of total variance and clustering accuracy was 100% between WT and *Ube3a*^*m-/p+*^ non-drug control groups when using 1 PC for clustering (Fig. S11b–d). CRISPR/Cas9 treatment resulted in an amelioration, but not full correction, of overall behavioral severity in *Ube3a*^*m-/p+*^ mice as measured by PC1 (Fig. 6b, c; main effect of group: *F*_(2,30)_ = 93.66, *p* < 0.0001; post hoc WT/control vs. AS/control: *p* < 0.0001; post hoc AS/control vs. AS/treatment: *p* < 0.0001). We observed a significant effect of treatment using PC1 under multiple conditions where different combinations of behavioral measures were included in the analysis (Fig. S11e–g).

### PUMBAA: A graphical user interface for multidimensional analysis of behavior

Multidimensional analysis may be valuable for other behavioral datasets in *Ube3a*^*m-/p+*^ mice and mouse models of related disorders. To enable widespread use of multidimensional behavioral analysis, we developed a graphical user interface for phenotyping using a multidimensional behavioral analysis algorithm (PUMBAA; Fig. S12; https://github.com/sidorovlab/PUMBAA). PUMBAA runs in a MATLAB environment but does not require users to have prior MATLAB coding knowledge. PUMBAA enables user control of analysis parameters for all steps including data selection, data standardization, principal component analysis, clustering, and validation.

## Discussion

Multidimensional analysis of behavior (Fig. 1) correctly predicted *Ube3a* genotype in a mouse model of AS with high accuracy (Fig. 2). This approach retained high accuracy with multiple combinations of behavioral data (Fig. 3) and in behavioral cohorts of a manageable size (*n* = ~12–15; Fig. 4). Principal component analysis enabled the simplification of an animal’s overall behavioral profile to a single severity score (PC1, Fig. 5) that demonstrated improvement following treatment (Fig. 6). We propose that multidimensional behavioral analysis provides a generalizable approach to quantify behavioral impairment and screen treatments preclinically in rodent models of neurodevelopmental disorders.

In the context of AS, the primary value of multidimensional behavioral analysis is to assess the efficacy of treatments in rodent models. Multiple promising treatments for AS are currently under development at various stages of clinical and preclinical testing [23, 43]. Current and future treatments will span multiple mechanisms of action, including directly targeting *Ube3a* expression, targeting downstream *Ube3a* protein targets, and more generalized symptom-based approaches. Simplifying mouse behavior to an overall severity score will be valuable for measuring overall improvement following treatment, and for assessing the effect of treatment across development. Our analysis found that each behavioral measure in the well-established Sonzogni battery [24] contributes roughly equally to PC1 (loadings range from 0.22–0.42; removal of single measures resulted in 92.3–96.4% accuracy), and that different combinations of behavioral inputs can be used to achieve high accuracy (Figs. 3 and 5a, b). These results suggest that multidimensional behavioral analysis can generalize well across different behavioral batteries and across different laboratories. In addition, multidimensional analysis is a tool that can generalize across mouse strains, mouse lines, and across species to be applied to the new *Ube3a*^*m-/p+*^ rat model [47]. Our study assessed behavior using the *Ube3a*^*m-/p+*^ mouse developed by Jiang and colleagues [34], which has been the most commonly used preclinical model for AS research. However, a limit to this mouse line is that it mimics the loss of UBE3A but not other nearby genes that are also deleted in the majority of individuals with AS. In humans, AS clinical severity is typically greater in individuals with a deletion genotype [48]. Multidimensional analysis can be used in the future to test whether AS mouse models with larger deletions [49] have a more severe behavioral phenotype than the *Ube3a*^*m-/p+*^ model.

Multidimensional analysis revealed that overall behavioral severity was improved but not fully corrected by paternal *Ube3a* unsilencing at E15.5 + P1. Incomplete behavioral improvement is consistent with the results of Wolter et al. on certain individual tests (e.g., rotarod, brain weight), though they did report full correction of impairments in hindlimb clasping [41]. We hypothesize that overall behavioral rescue was incomplete due to the amount of UBE3A reinstatement achieved using CRISPR/Cas9-based unsilencing: Wolter et al. achieved reinstatement of UBE3A protein to ~40% of WT levels in a subset of animals where Western blotting was performed [41]. An advantage of multidimensional analysis is that for future treatment studies, PC1 (as a readout of overall behavioral severity) can be correlated with the degree of UBE3A reinstatement achieved within individual animals.

Beyond AS, this study provides proof of concept that multidimensional behavioral analysis can be applied to rodent models of related disorders. Prior studies have generally applied principal component analysis to rodent behavioral data in two contexts: (a) to assess which subset of behavioral measures are most relevant or valuable [14, 17, 18], and (b) to attempt to categorize two or more groups based on their behavior [15, 16, 22]. Here, we applied both approaches to quantify behavior in *Ube3a* mutants. Multidimensional analysis was especially effective in *Ube3a*^*m-/p+*^ mice because behavioral impairments on individual tests are so reliable and widespread in this line [5]. However, behavioral phenotypes in other lines are often less robust. We hypothesize that multidimensional analysis will be particularly valuable for detecting subtle behavioral differences in established mouse models and for screening behavior in new mouse models of rare disorders. Multidimensional analysis used in this context is unlikely to result in false positive effects of genotype (Fig. S7). To enable widespread use of multidimensional behavioral analysis, we developed a graphical user interface (PUMBAA; Fig. S12) to simplify and generalize analysis methods. PUMBAA enables users to perform customized multidimensional analysis in a MATLAB environment without any prior coding knowledge.

Our work identified a number of practical considerations for future studies using multidimensional analysis to quantify behavior in rodent models of NDDs. First, multidimensional analysis can only be performed in datasets where longitudinal behavioral testing is performed within animals. This requirement places certain limits on experimental design, such as the inclusion of tests that may be terminal (e.g., audiogenic seizures). Longitudinal testing also presents challenges when assessing the efficacy of treatment. For example, a four-week longitudinal behavioral battery would not be appropriate for a treatment expected to last two weeks. In addition, accounting for sex differences is an important consideration for multidimensional analysis, as sex differences in behavior have been reported in *Ube3a*^*m-/p+*^ mice and in rodent models of related disorders (Fig. S2) [24, 45, 50–53]. Accounting for sex differences in behavior resulted in a slight but not statistically meaningful improvement in the accuracy of multidimensional analysis from ~91% to ~95% (Figs. 2 and S6). Finally, our results suggest that cross-cohort behavioral variability decreases the accuracy of multidimensional analysis performed across multiple behavioral cohorts (Fig. 4). The inclusion of behavioral assessments with no group differences would not “dilute” the effectiveness of PCA; thus, multidimensional analysis is well-suited for analysis of broad behavioral phenotyping regimens [12, 13]. Future studies using multidimensional analysis of behavior may also consider additional methods (beyond percent accuracy) to quantify the degree to which two genotypes can be distinguished in PC space. Other potentially valuable parameters include the distance between cluster centroids and the spread of data within individual clusters.

Overall, multidimensional behavioral analysis enables representation of behavior in *Ube3a*^*m-/p+*^ mice as a single severity score that is reliably different from wild-type controls and is sensitive to treatment. Multidimensional behavioral analysis represents a tool that may be used to evaluate the effectiveness of preclinical treatments for AS and related neurodevelopmental disorders.

## Supplementary information

Supplemental Material
