# Supplementary material for: Multidimensional analysis of behavior predicts genotype with high accuracy in a mouse model of Angelman syndrome
Source: Transl Psychiatry. 2022 Oct 3;12:426. doi: 10.1038/s41398-022-02206-3 (PMC9529912; doi:10.1038/s41398-022-02206-3)
Supplement: Supplementary file 1 — Supplemental Material [file 41398_2022_2206_MOESM1_ESM.pdf]

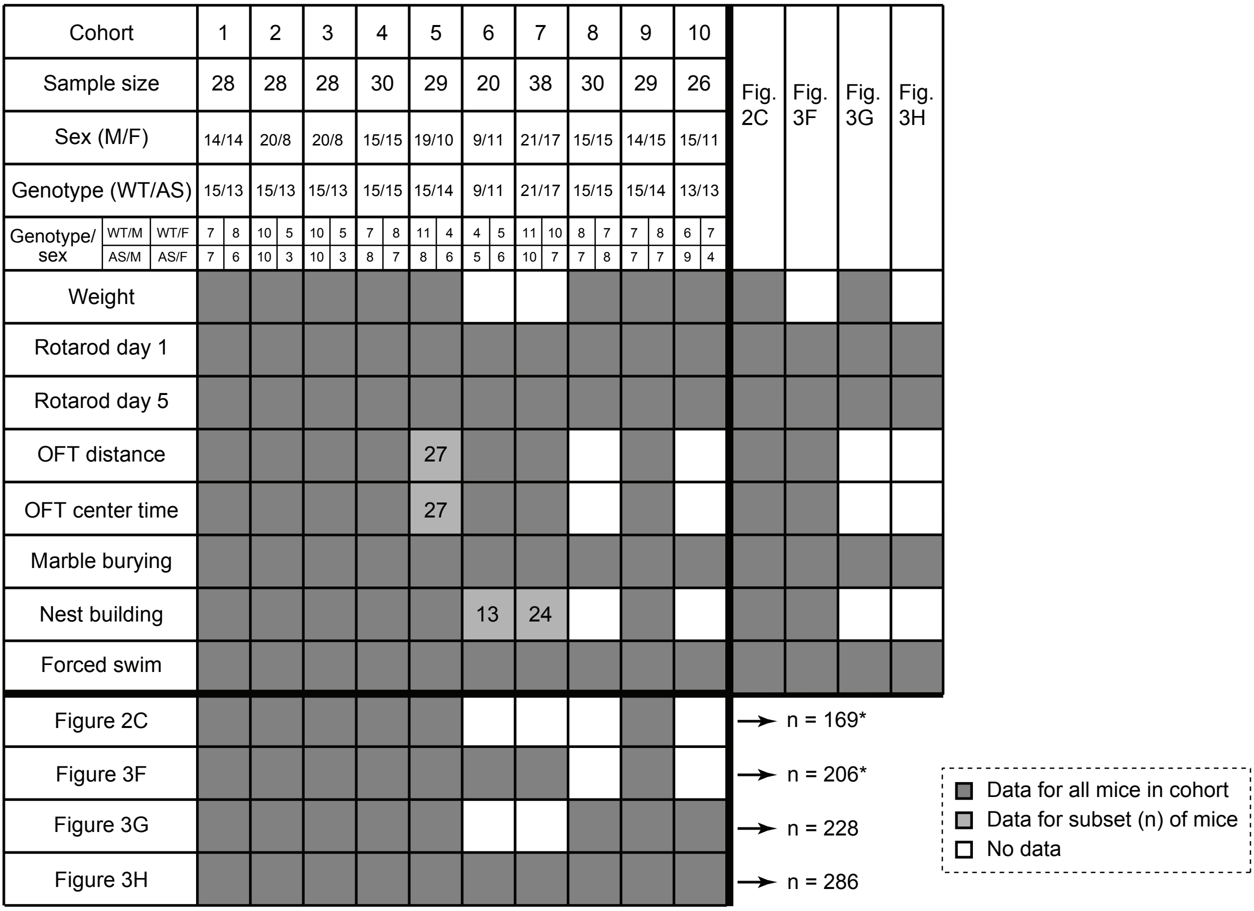

Supplementary Figure 1: Overview of experimental cohorts and behavioral tests performed. Ten total cohorts were used for this study. All behavioral tests were performed in six of ten cohorts (1-5, 9). Cohorts 1-8 were previously reported by Sonzogni and colleagues[24] and cohorts 9-10 represent new data. Boxes in light gray indicate missing data for a subset of animals on one test within a cohort (e.g. open field data was absent for 2/29 animals in cohort 6). Asterisks in total *n* indicate removal of one outlier for multidimensional analysis. Cohorts 1-5 and 9 were used for Figs. 2, 3a-d, 4a, 5, S3-S6, S8a, S8c, S10. Cohorts 1-7 and 9 were used for Fig. 3e and 3f. Cohorts 1-5 and 8-10 were used for Fig. 3e and 3g. Cohorts 1-10 were used for Figs. 1, 3e, 3h, and S2.

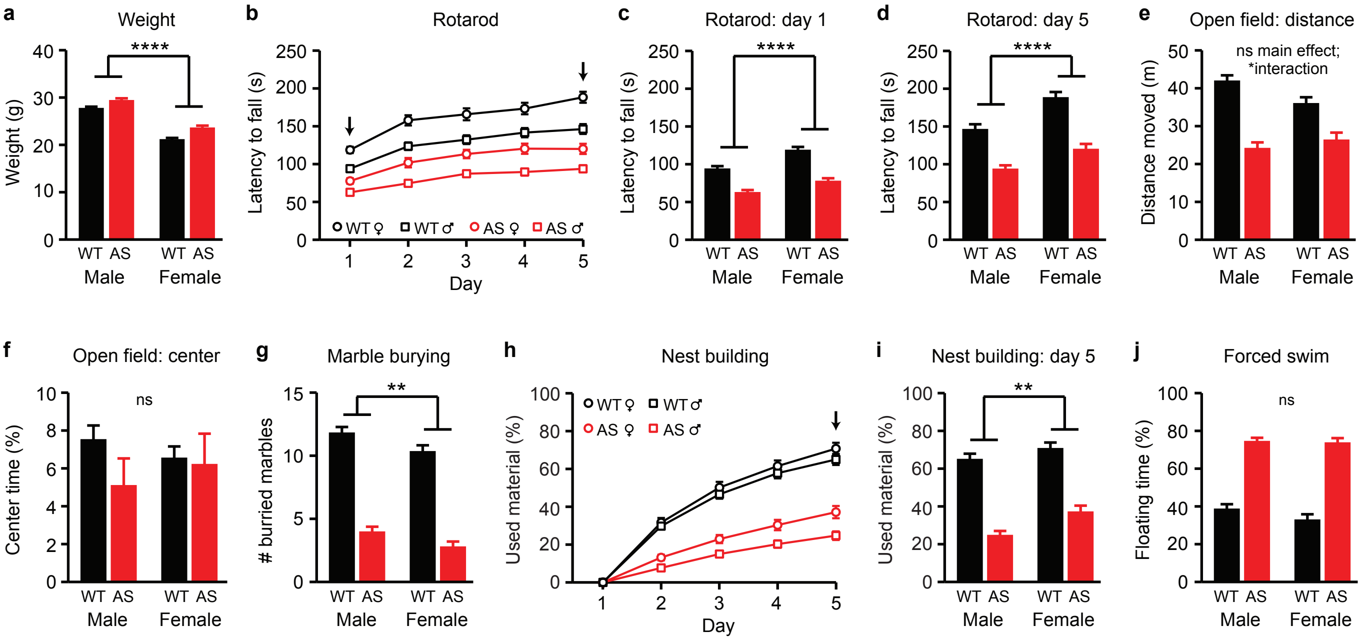

k

Sample size

Group	Panel S2a	S2b	S2c	S2d	S2e	S2f	S2g	S2h	S2i	S2j
WT, female	52	67	67	67	51	51	67	50	50	66
WT, male	66	81	81	81	67	67	81	59	59	82
AS, female	44	57	57	57	45	45	57	40	40	57
AS, male	66	81	81	81	65	65	81	60	60	81

Supplementary Figure 2: Assessment of behavioral performance by sex. Black: WT, red: *Ube3a*$^{m-/p+}$ (AS). (a) Weight: there was a significant main effect of sex ($F_{(1,224)} = 414.0$, $p < 0.0001$) and no interaction between sex and genotype ($F_{(1,224)} = 1.747$, $p = 0.1876$). Post hoc tests revealed significant WT-AS differences in both male and female mice ($p < 0.0001$). (b) Overall rotarod performance: there was a significant main effect of sex ($F_{(1,282)} = 38.25$, $p <0.0001$) and no interaction between sex and genotype ($F_{(1,224)} = 1.747$, $p = 0.1876$). Arrows indicate measures used for multidimensional analysis and analyzed separately in panels c-d. (c) Day 1 rotarod performance: there was a significant main effect of sex ($F_{(1,282)} = 28.68$, $p < 0.0001$) and no interaction between sex and genotype ($F_{(1,282)} = 1.768$, $p = 0.1847$). Post hoc tests revealed significant WT-AS differences in both male and female mice ($p < 0.0001$). (d) Day 5 rotarod performance: there was a significant main effect of sex ($F_{(1,282)} = 89.99$, $p < 0.0001$) and no interaction between sex and genotype ($F_{(1,282)} = 1.531$, $p = 0.2171$). Post hoc tests revealed significant WT-AS differences in both male and female mice ($p < 0.0001$). (e) Total distance traveled on the open field test: there was no significant main effect of sex ($F_{(1,224)} = 1.328$, $p = 0.2503$). However, there was a significant interaction between sex and genotype ($F_{(1,224)} = 6.223$, $p = 0.0133$). Post hoc tests revealed significant male-female differences in WT ($p = 0.0179$) but not AS ($p = 0.5822$) mice. (f) Center time on the open field test: there was no significant main effect of sex ($F_{(1,224)} = 0.003305$, $p = 0.9542$) or sex X genotype interaction ($F_{(1,224)} = 0.8029$, $p = 0.3712$). (g) Marble burying: there was a significant main effect of sex ($F_{(1,282)} = 8.504$, $p = 0.0038$) and no interaction between sex and genotype ($F_{(1,282)} = 0.09829$, $p = 0.7541$). Post hoc tests revealed significant WT-AS differences in both male and female mice ($p < 0.0001$). (continued on next page)

Supplementary Figure 2 (legend continued)

(h) Overall nest building performance: there was a significant main effect of sex ($F_{(1,205)}$ = 7.441, $p = 0.0069$) and no interaction between sex and genotype ($F_{(1,205)}$ = 1.263, $p = 0.2623$). Arrows indicate measure used for multidimensional analysis and analyzed separately in panel i. (i) Day 5 (total) nest building: there was a significant main effect of sex ($F_{(1,205)}$ = 9.954, $p = 0.0018$) and no interaction between sex and genotype (interaction $F_{(1,205)}$ = 1.325, $p = 0.2511$). Post hoc tests revealed significant WT-AS differences in both male and female mice ($p < 0.0001$). (j) Forced swim: there was no significant main effect of sex ($F_{(1,282)}$ = 1.786, $p = 0.1825$) or sex X genotype interaction ($F_{(1,282)}$ = 1.013, $p = 0.3149$). (k) Sample sizes for data summarized by each figure panel. Data represent mean ± SEM; *$p < 0.05$, **$p < 0.01$, ****$p < 0.0001$. Asterisks on graphs indicate main effect of sex, except *interaction where noted.

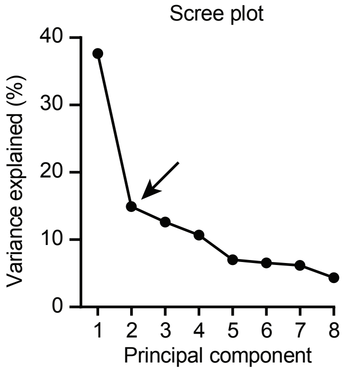

Supplementary Figure 3: Two principal components capture the majority of variance in the dataset. A Scree plot illustrates that PC2 serves as the "elbow" cutoff point beyond which inclusion of additional PCs will likely provide limited benefit.

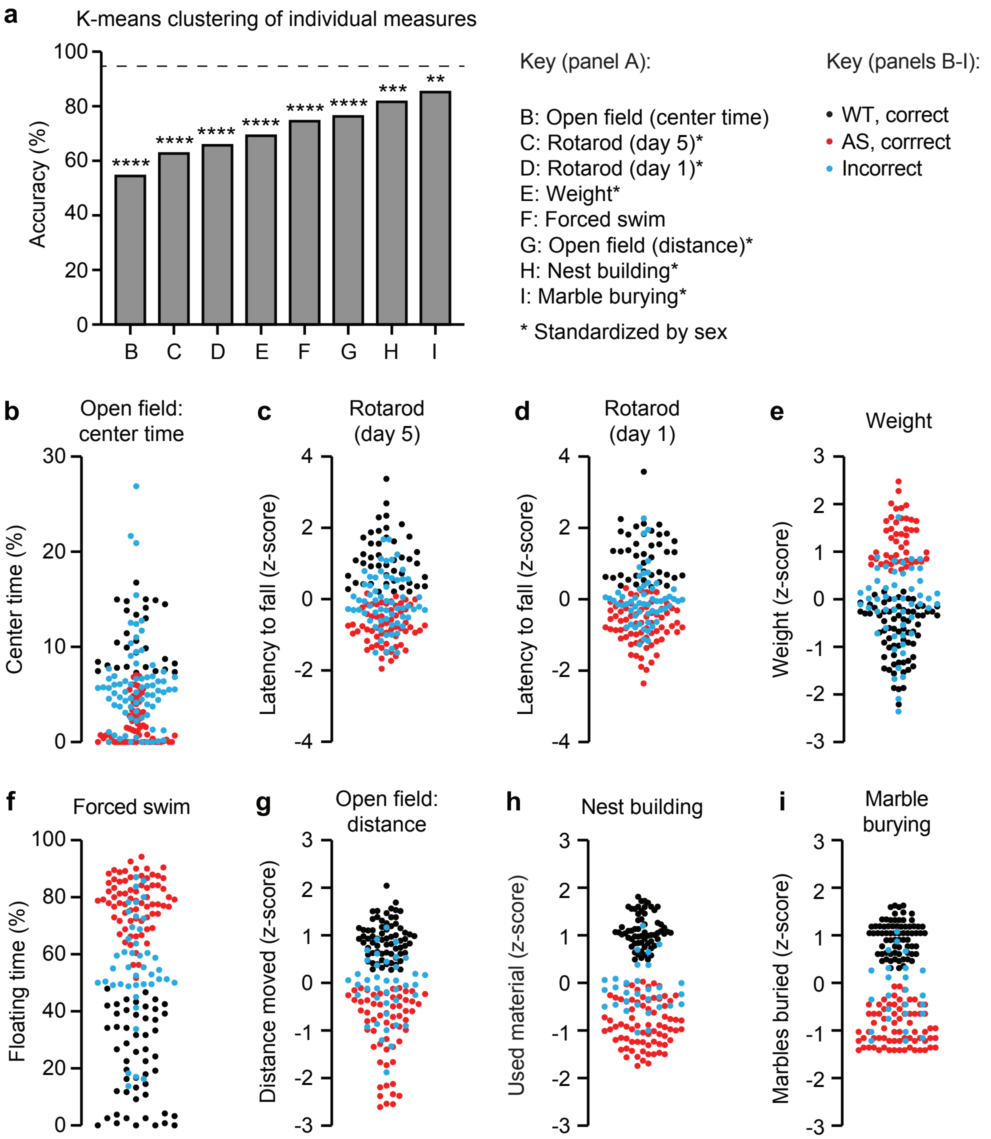

Supplementary Figure 4: Clustering using performance on individual behavioral measures is less effective than clustering in principal component space. (a) Summary of k-means clustering of each behavioral measure. Clustering accuracy of individual measures ranged from 54.4% (open field center time) to 85.2% (marble burying). For measures that were sex-dependent, k-means clustering was performed after standardization to account for sex. The dashed line represents the 94.7% accuracy from multidimensional analysis of behavior (Fig. 2c). Asterisks indicate statistically meaningful differences from clustering accuracy using PCA + k-means clustering (Fisher's exact test; $^{**}p < 0.01$, $^{***}p < 0.001$ $^{****}p < 0.0001$). (b-i) Accuracy of clustering for each individual behavioral measure (black = WT, correct; red = AS, correct, blue = incorrect).

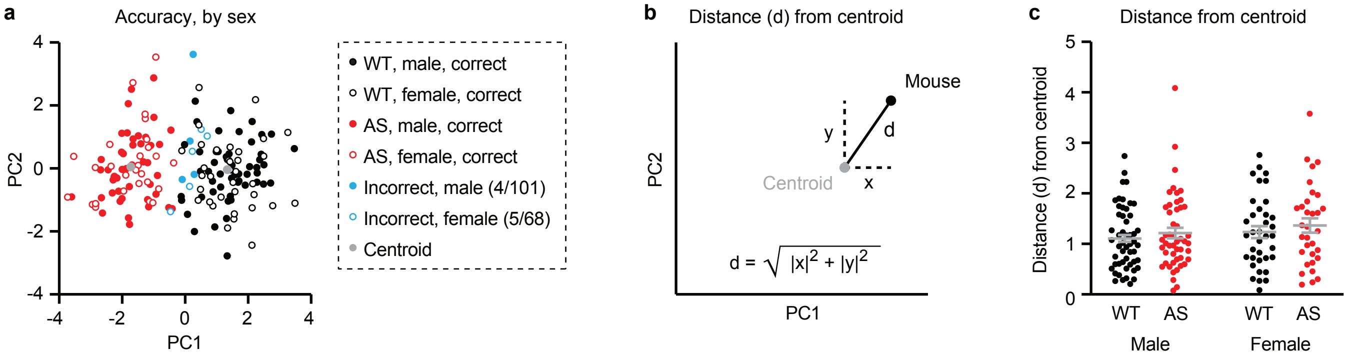

Supplementary Figure 5: Male and female mice are equally distributed in principal component space. (a) Data from Figure 2a are re-plotted, with male and female mice indicated by closed and open circles. Centroids of clusters are illustrated with gray closed circles. (b) Schematic illustrating calculation of the distance from the centroid, used for every mouse in PC space. (c) Summary of distances from centroid for all animals. There was no difference in the distance from cluster centroid between male and female mice (two-way ANOVA, main effect of sex: $F_{(1,165)} = 1.541$, $p = 0.2163$). Data points shown here correspond to the actual sex of animals, and incorrect classifications are included in analysis. WT/male: $n = 52$, AS/male: $n = 49$, WT/female: $n = 36$, AS/female: $n = 32$.

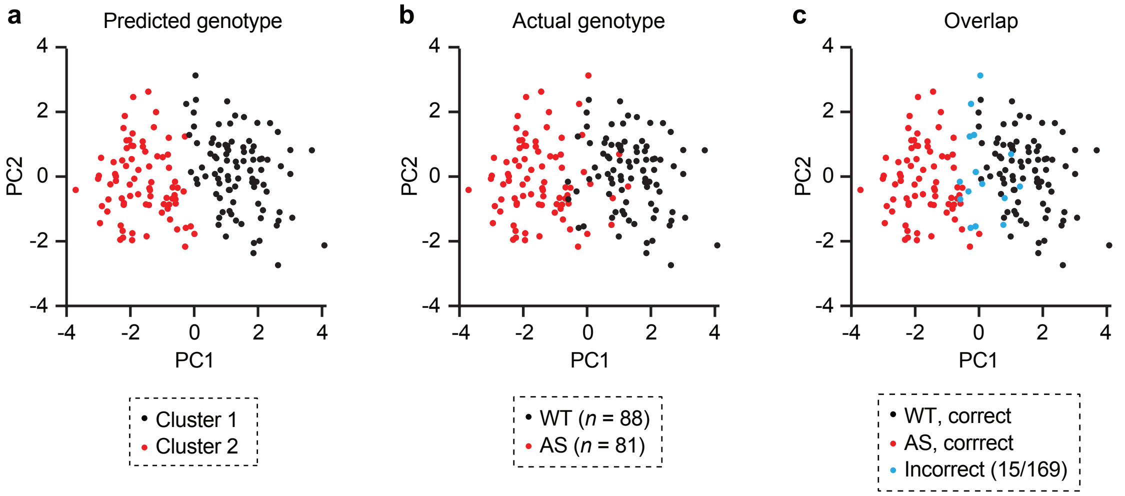

Supplementary Figure 6: Assessing the accuracy of multidimensional analysis without accounting for sex differences in behavior. (a) PCA plus k-means clustering categorized two clusters, with each dot representing one mouse's behavioral profile. Data were not standardized separately by sex for this analysis. (b) Actual genotype of animals (black: WT, red: AS). (c) An overlay of panels A and B with animals clustered incorrectly labeled in blue. Clustering accuracy was 91.1% without accounting for sex (and 94.7% with accounting for sex). The difference between these conditions is not statistically significant (Fisher's exact test, $p = 0.2896$). PC1 + PC2 accounted for 53.8% of total variance.

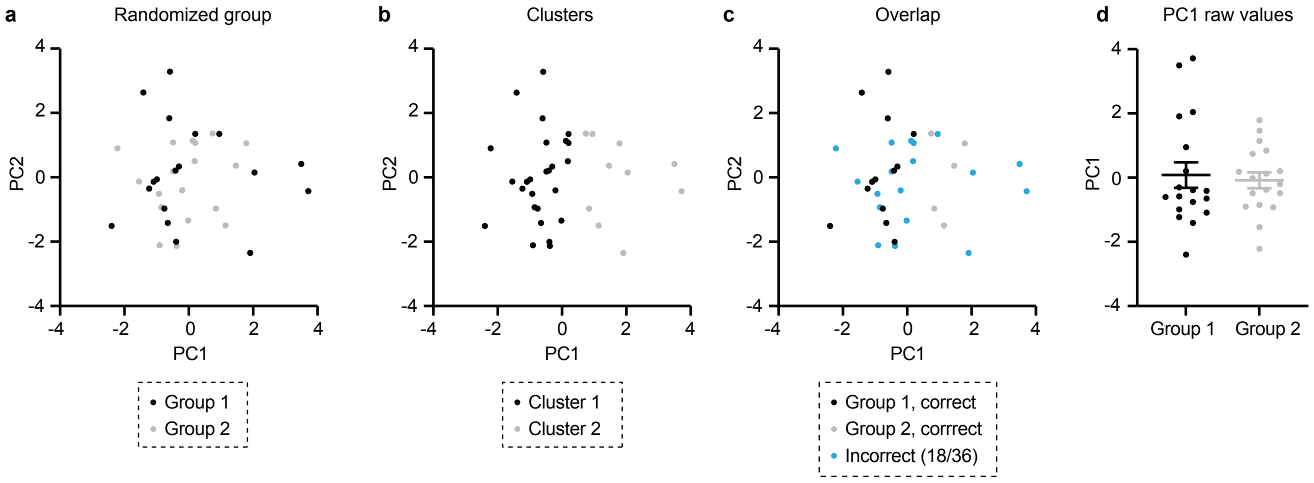

Supplementary Figure 7: Multidimensional analysis does not detect false positive group effects. All wild-type females in Dataset 1 ($n = 36$) were used for this analysis. Subjects were randomly assigned into two groups to simulate a dataset where there are two genotypes but no genotype differences in behavior. (a) Behavioral data plotted in 2PC space by group. (b) k-means clustering of animals in 2PC space. (c) 50% of animals (18/36) were classified correctly based on their behavioral profile. (d) There was no statistical difference in PC1 between groups ($t_{(34)} = 0.3568$, $p = 0.7235$). Together, this analysis demonstrates that multidimensional analysis does not artificially pull out group differences in behavior when no differences are present.

a Variance explained (Figure 3a)

Measure(s) removed	Variance explained by PC1 + PC2
W: Weight	57.6%
NB: Nest building	54.1%
FS: Forced swim	54.3%
OFD: Open field (distance)	52.8%
OFC: Open field (center time)	56.1%
OFA: Open field (all)	60.1%
MB: Marble burying	54.1%
NR: Nothing removed	52.6%
R1: Rotarod (day 1)	55.3%
R5: Rotarod (day 5)	57.1%
RA: Rotarod (all)	61.9%

b Variance explained (Figure 3e)

Condition	Variance explained by PC1 + PC2
8 measures, 6 cohorts (2c)	52.6%
7 measures, 8 cohorts (3e)	57.3%
5 measures, 8 cohorts (3f)	67.2%
4 measures, 10 cohorts (3g)	76.6%

c Variance explained (Figure 4a, PC1 + PC2)

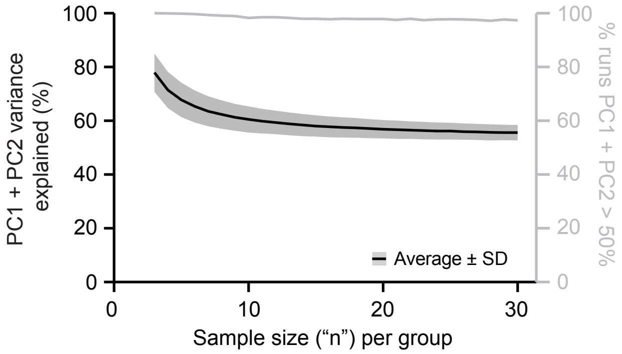

Supplementary Figure 8: Two principal components account for a majority of the variance for the analyses used in Figures 3 and 4. (a) With one measure or one behavioral test removed from analysis (Fig. 3a), the variance explained by PC1 + PC2 ranged from 52-62%. (b) Using four different conditions for data inclusion (Fig. 3e), the variance explained by PC1 + PC2 ranged from 52-77%. (c) Across the entire bootstrap analysis (Fig. 4a), the variance explained by PC1 + PC2 remained consistently above 50%. The black line represents the average variance explained by PC1 + PC2 across all 10,000 runs (±SD), and the gray line represents the percentage of runs where PC1 + PC2 exceeded 50% of total variance.

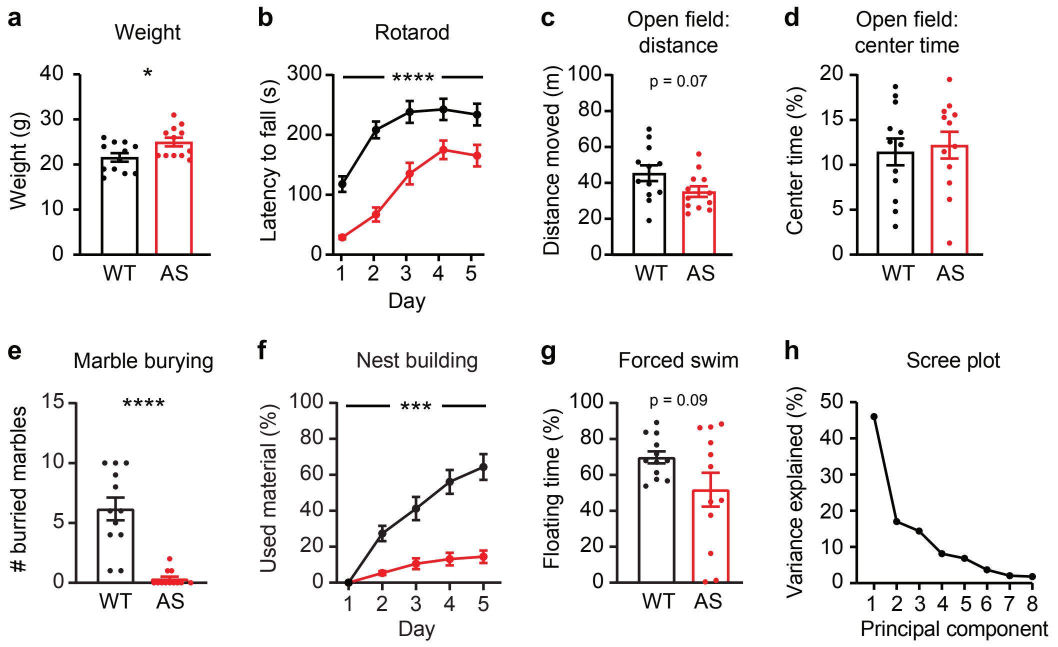

Supplementary Figure 9: Raw behavioral results in new cohort of *Ube3a*$^{m-/p+}$ mice. Data plotted from Dataset 2. Black: WT (n = 12), red: *Ube3a*$^{m-/p+}$ (AS; n = 12). (a) Weight was increased in *Ube3a*$^{m-/p+}$ mice (Student's t-test; $t_{(22)} = 2.519$, $*p = 0.0196$). (b) Rotarod: there was a significant main effect of genotype (two-way RM ANOVA: $F_{(1,22)} = 32.32$, $****p < 0.0001$) and a significant time X genotype interaction ($F_{(4,88)} = 3.616$, $p = 0.0089$). (c) Open field distance was not significantly different by group ($t_{(22)} = 1.935$, $p = 0.0660$). (d) Open field center time was not significantly different by group ($t_{(22)} = 0.3560$, $p = 0.7252$). (e) Marble burying was decreased in *Ube3a*$^{m-/p+}$ mice ($t_{(22)} = 6.010$, $****p < 0.0001$). (f) Nest building was impaired in *Ube3a*$^{m-/p+}$ mice (main effect of genotype: $F_{(1,22)} = 29.02$, $****p < 0.0001$; time X genotype interaction: $F_{(3,66)} = 26.16$, $p < 0.0001$). (g) Forced swim was not significantly different by group ($t_{(22)} = 1.797$, $p = 0.0861$). (h) Scree plot illustrating the amount of variance explained by each PC for Dataset 2; PC1 + PC2 = 63.0%.

a

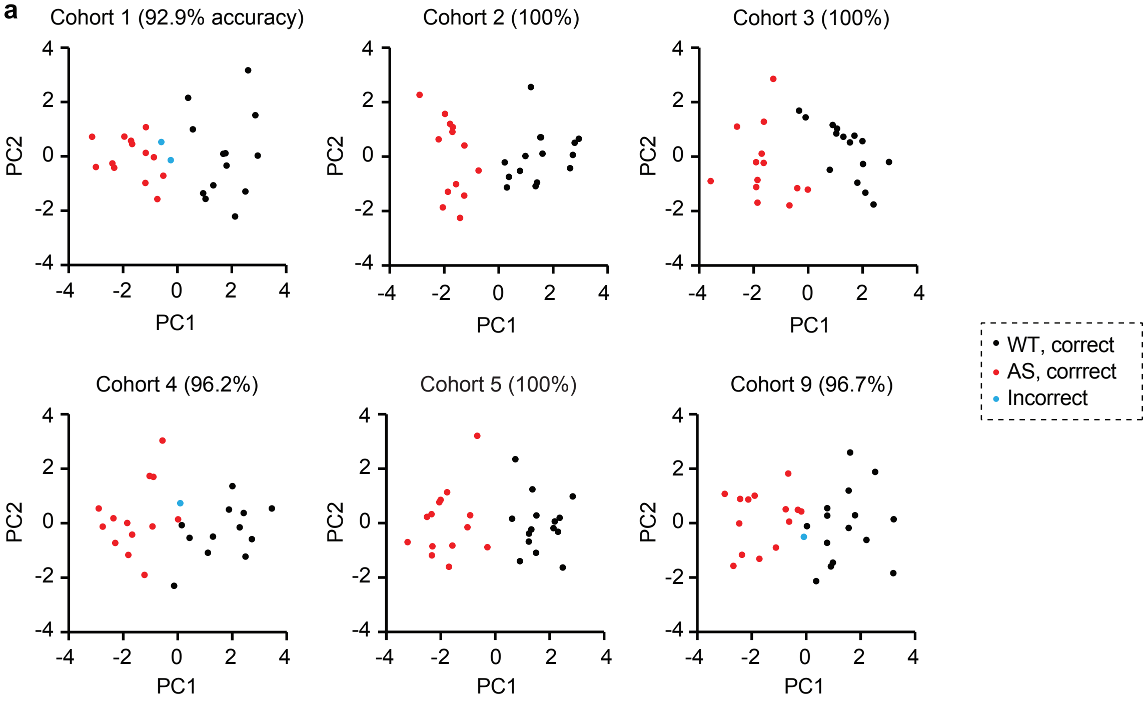

b

Variance explained, panel (a)

Dataset 1 cohort	Variance explained by PC1 + PC2
Cohort 1	59.2%
Cohort 2	59.5%
Cohort 3	59.0%
Cohort 4	59.2%
Cohort 5	62.5%
Cohort 9	61.5%

Supplementary Figure 10: Multidimensional analysis predicts Ube3a genotype with high accuracy in each of six behavioral cohorts. Raw data accompanying Figure 4a. The clustering accuracy for each of six independent cohorts ranges from 92.9% to 100% and corresponds to an open circle in Figure 4a.

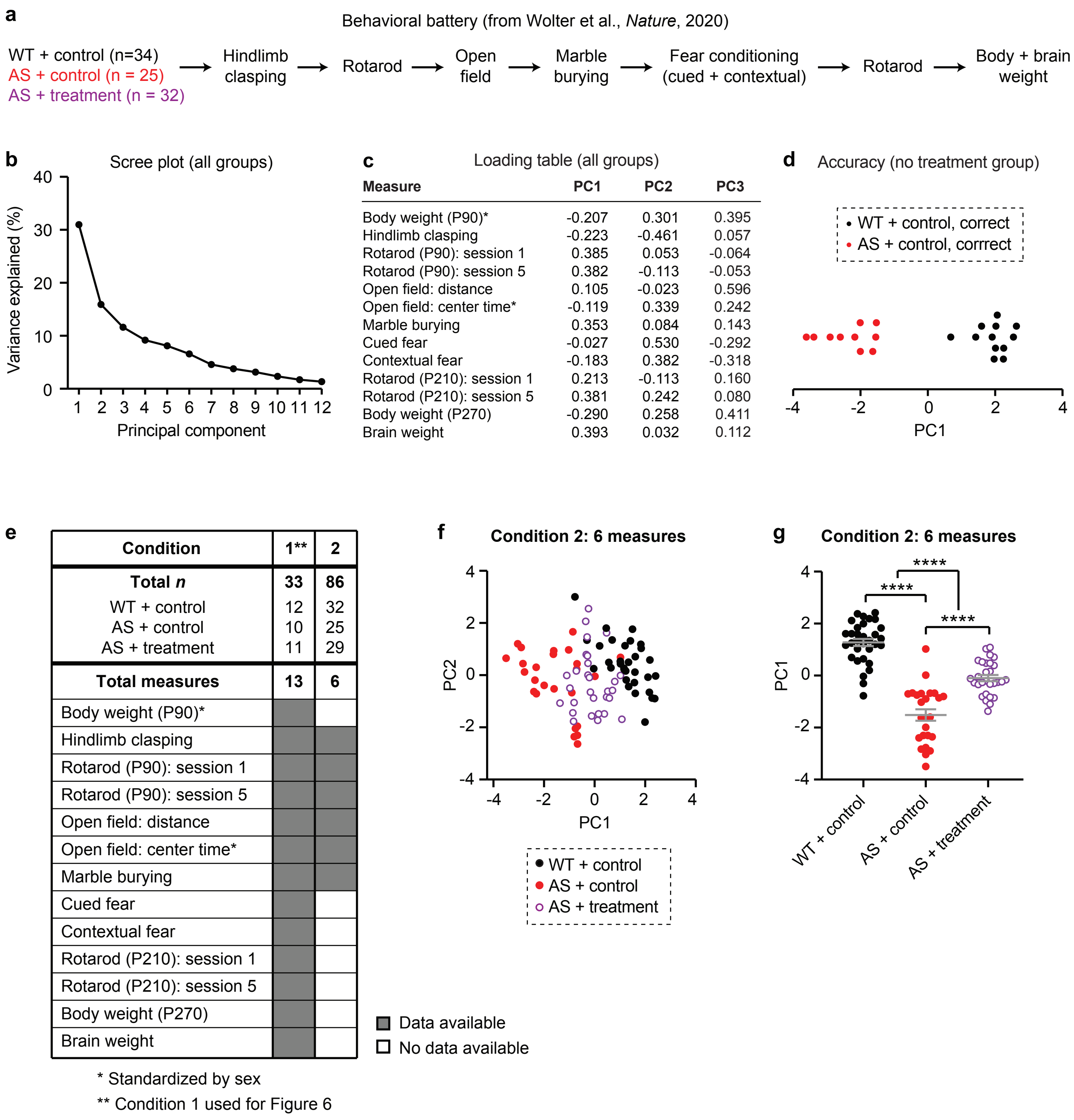

Measure	PC1	PC2	PC3
Body weight (P90)*	-0.207	0.301	0.395
Hindlimb clasping	-0.223	-0.461	0.057
Rotarod (P90): session 1	0.385	0.053	-0.064
Rotarod (P90): session 5	0.382	-0.113	-0.053
Open field: distance	0.105	-0.023	0.596
Open field: center time*	-0.119	0.339	0.242
Marble burying	0.353	0.084	0.143
Cued fear	-0.027	0.530	-0.292
Contextual fear	-0.183	0.382	-0.318
Rotarod (P210): session 1	0.213	-0.113	0.160
Rotarod (P210): session 5	0.381	0.242	0.080
Body weight (P270)	-0.290	0.258	0.411
Brain weight	0.393	0.032	0.112

Condition	1**	2
**Total *n***	**33**	**86**
WT + control	12	32
AS + control	10	25
AS + treatment	11	29
**Total measures**	**13**	**6**
Body weight (P90)*	■	
Hindlimb clasping	■	■
Rotarod (P90): session 1	■	■
Rotarod (P90): session 5	■	■
Open field: distance	■	■
Open field: center time*	■	■
Marble burying	■	■
Cued fear	■	
Contextual fear	■	
Rotarod (P210): session 1	■	
Rotarod (P210): session 5	■	
Body weight (P270)	■	
Brain weight	■	

* Standardized by sex

** Condition 1 used for Figure 6

Supplementary Figure 11: CRISPR/Cas9-mediated paternal *Ube3a* unsilencing improves overall behavior in *Ube3a*$^{m-/p+}$ mice. (a) Experimental timeline, as described and performed by Wolter et al., Nature (2020) (Dataset 3). (b) Scree plot showing the variance in Dataset 3 (Fig. 6) explained by each principal component. PC1 + PC2 accounts for 46.9% of total variance in this dataset. (c) Loadings for PCA perfomed on Dataset 3 (Fig. 6) demonstrate that the majority of individual behaviors remain strongly correlated with PC1. (d) Multidimensional analysis performed on only the WT + control and *Ube3a*$^{m-/p+}$ + control groups results in 100% clustering accuracy in 1PC space. (continued on next page)

Supplementary Figure 11 (legend continued)

(e) Overview of behavioral tests performed in different subsets of animals. All behavioral measures were performed in 33 of 86 total mice (Condition 1). Six of 13 measures were performed in all 86 mice (Condition 2). Condition 1 was used for multidimensional analysis in Figure 6. (f) Multidimensional analysis under Condition 2; data plotted in 2PC space. (g) Multidimensional analysis under Condition 2 using 1 PC reveals amelioration of behavioral impairments in AS + treatment group (one-way ANOVA: $F_{(2,83)} = 76.46$, $p < 0.0001$; post hoc WT/control vs. AS/control: ****$p < 0.0001$; post hoc AS/control vs. AS/treatment: ****$p < 0.0001$). Five animals were included in behavioral testing (panel a) but not in either condition 1 or condition 2 of multidimensional analysis (panel e). These animals did not have open field testing and marble burying. Data represent mean ± SEM; ****$p < 0.0001$.

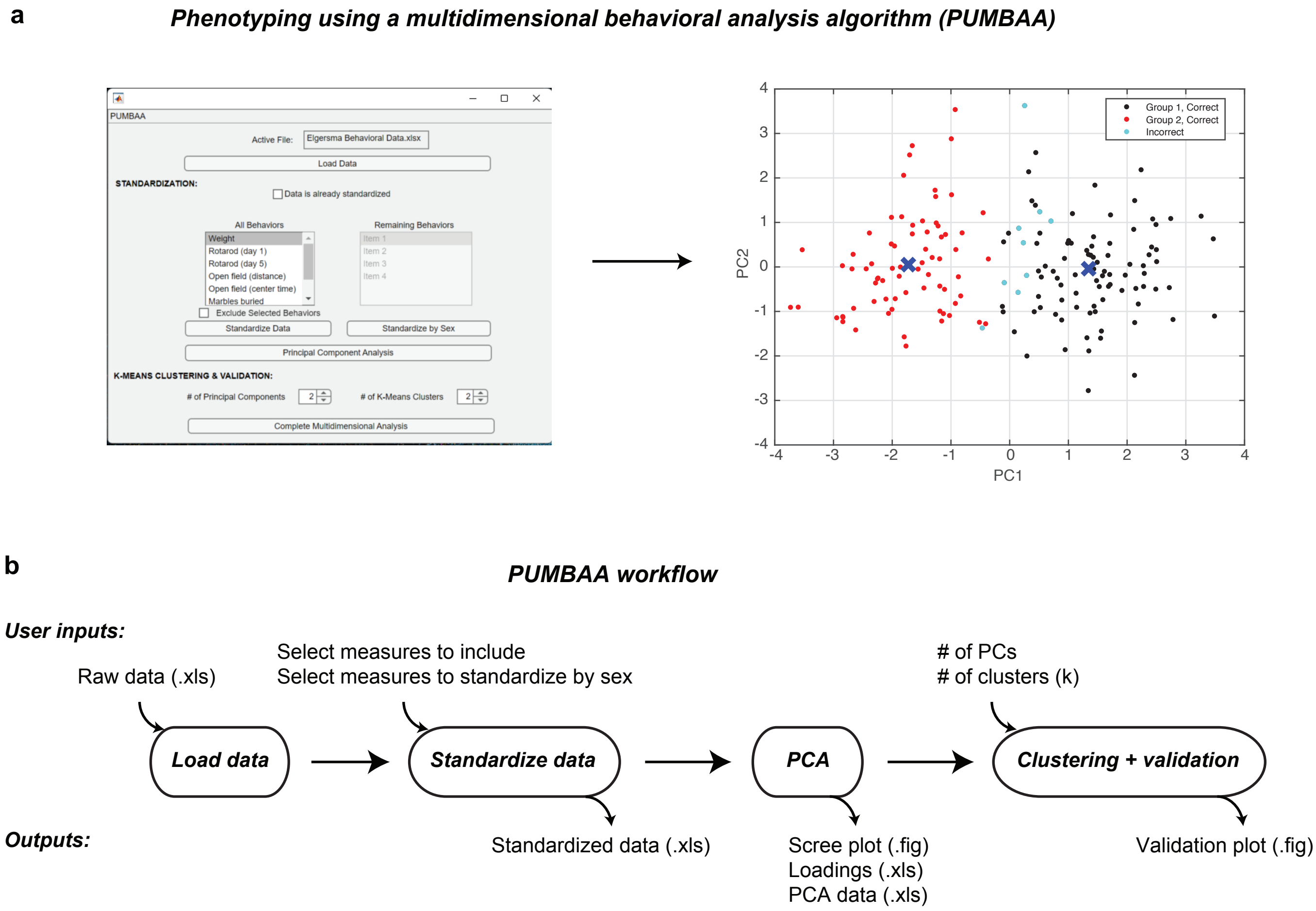

Supplementary Figure 12: PUMBAA: a graphical user interface for phenotyping using a multidimensional behavioral analysis algorithm. (a) Screen shots of main PUMBAA interface and main validation plot generated by the program. (b) Schematic illustrates main analysis steps, the user inputs required for each step, and the outputs generated by the program following each step.
